# Aspirin in primary prevention: USPSTF recommendations

**Published:** 2010-06

**Authors:** J Aalbers

**Affiliations:** Special Assignments Editor

## Introduction

Recent clinical studies such as the Japanese Primary Prevention trial (JPAD)^1^ and the Aspirin for Asymptomatic Atherosclerosis study (AAA)^2^ have resulted in considerable debate on when and for whom aspirin should be given as primary prevention for vascular events.

The US Preventative Services Task Force (USPSTF) has recently updated its recommendations from new evidence on the benefit and harm of aspirin for the primary prevention of cardiovascular disease, including myocardial infarction and stroke.^3^ All their recommendations are allocated a graded level to indicate the extent of data available to support the advocated approach in both adult men and women without a history of coronary artery disease or stroke.

In men, the USPSTF recommends the use of aspirin for men aged 45 to 79 years when the potential benefit due to a reduction in myocardial infarctions outweighs the potential harm due to an increase in gastrointestinal bleeding. In younger men (45–59 years), the benefits outweigh the increased bleeding at a 10-year coronary heart disease (CHD) risk of greater than 4%. In the older age group, under 80 years, the benefit–risk ratio is at 9 to 12% of the 10-year cardiovascular risk as measured by a Framingham Heart studyderived risk-assessment tool.^4^

In women, the USPSTF recommends the use of aspirin for women aged 55 to 79 years when the potential benefit of a reduction in ischaemic strokes outweighs the bleeding risk. In women aged 55 to 59 years, estimated harm is balanced by benefit at a 2% 10-year stroke risk; which rises to 11% in the age group 60 to 69 years and 17% in the under 80-year-old group. Table 1 summarises these benefit– risk balances.

**Table 1:** 

*Men*		*Women*	
*Age (years)*	*10-year CHD risk (%)*	*Age (years)*	*10-year stroke risk (%)*
45–59	≥ 4	55–59	≥ 3
60–69	≥ 9	60–69	≥ 8
70–79	≥ 12	70–79	≥ 11

Adapted from Ann Intern Med 2009; 150(6): 396–404.

A key issue for the practising clinician is when to recommend against taking aspirin. In an editorial in the Annals of Internal Medicine,^5^ Mehta pointed out that the rule of benefit outweighing risk assumes that patients place the same value on avoiding a bleeding event as they do on avoiding a stroke or myocardial infarction. Depending on where the bleeding occurs, some patients would rather avoid a stroke than avoid a bleeding event and would therefore prefer to take aspirin. Discussing benefits and risks with the individual patient is therefore essential. Clearly also, patients at relatively high risk for intracranial bleeding should absolutely avoid aspirin.

Aspirin is still underused and these USPSTF recommendations should assist clinicians to extend the benefits of aspirin to more patients.
